# Silent Killer: Case Report of Acute Gastrostomy Tube Erosion

**DOI:** 10.5811/westjem.2015.1.25321

**Published:** 2015-02-26

**Authors:** Allen D. Chang, Darshan Thota, James M. Liang

**Affiliations:** Naval Medical Center San Diego, Department of Emergency Medicine, San Diego, California

An 87-year-old male with multiple medical problems and percutaneous endoscopic gastrostomy (PEG) tube placement presented to the emergency department for recurrent dysphagia, constipation, and concern for stool appearing in his PEG tube. The patient denied PEG tube complications over the past year. The patient’s vitals were within normal limits, and the exam was notable for a soft, non-tender, non-distended abdomen without masses or pain, with fecal contents observed in the PEG tube. Lab studies were unremarkable, and acute abdominal series films showed no evidence of obstruction or free air. A chest tomography (CT) of the abdomen and pelvis with contrast was performed, showing the gastrostomy tube linking the anterior aspect of the middle stomach with a portion within the transverse colon (Figures 1 and 2). The patient was admitted to the general surgery service for removal of the migrated PEG tube that eroded into the viscera.

Migration of PEG tubes into the colon is a rare complication of PEG tube insertion occurring in only 0.8% of patients in a seven-year clinical study.^1^ While uncommon, it is considered a major complication requiring hospital admission and specialty consultation, due to risk for further migration, peritonitis, or sepsis. Presenting symptoms of a colonic PEG migration include the onset of copious diarrhea and cramping (50%), often after tube feeding, fecal leakage (39%), or odorous fecal exudates from the stoma.^2^ Risk factors for migration and fistula formation include previous abdominal surgery, post-surgical adhesions, and a superiorly displaced transverse colon.

Assessment may be delayed due to overlying medical co-morbidities and otherwise unremarkable physical examination, which can delay accurate evaluation and surgical intervention. Of note, patients with neurological injuries may have limited perception of pain, resulting in reduced clinical flags potentially masking serious complications.^3^ Thus, close attention to vague abdominal or referred symptoms in this patient population is critical to timely diagnosis and treatment.

## Figures and Tables

**Figure 1:**
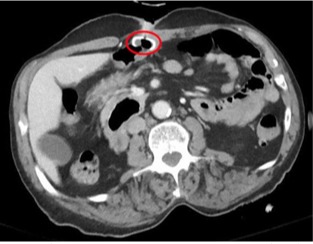
Cross section showing erosion into bowel wall (circle).

**Figure 2:**
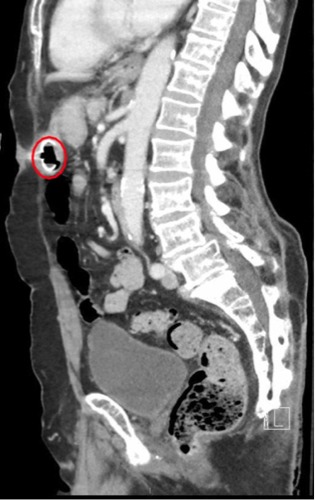
Sagittal section showing erosion into bowel wall (circle).
